# Supplementary material for: Effect of girls’ education on cancer awareness and screening in a natural experiment in Lesotho
Source: Nat Commun. 2025 Apr 20;16:3737. doi: 10.1038/s41467-025-58875-3 (PMC12009962; doi:10.1038/s41467-025-58875-3)
Supplement: Supplementary file 1 — Supplementary Information [file 41467_2025_58875_MOESM1_ESM.pdf]

SUPPLEMENTARY INFORMATION

Note S1. School-entry age policy 2

Note S2. Assumptions for causal inference 3

Note S3. Results for the subsample of men 4

Figure S1. Study participant flow diagram 5

Figure S2. Distribution of month of birth among women 6

Figure S3. Placebo outcome: measured adult height (in cm) by month of birth among women aged 25–49 years in the Lesotho DHS 2009-10 and 2014 7

Figure S4. Intention-to-treat results: cancer awareness and screening by month of birth among respondents aged 25–49 years in the Lesotho DHS 2009-10 and 2014 8

Figure S5. Intention-to-treat results: measured literacy, wealth and access to care by month of birth among women aged 25–49 years in the Lesotho DHS 2009-10 and 2014 9

Table S1. Intention-to-treat regression results for the relationship between being born after June 30th and year of schooling completed among women 10

Table S2. Sensitivity analyses: 2SLS regression results among women for breast cancer awareness when using alternative model and sample specifications 11

Table S3. Sensitivity analyses: 2SLS regression results among women for breast self-exam when using alternative model and sample specifications 12

Table S4. Sensitivity analyses: 2SLS regression results among women for breast clinical screening when using alternative model and sample specifications 13

Table S5. Sensitivity analyses: 2SLS regression results among women for cervical cancer awareness when using alternative model and sample specifications 14

Table S6. Sensitivity analyses: 2SLS regression results among women for cervical cancer screening when using alternative model and sample specifications 15

Table S7. Sensitivity analyses: using Poisson regression models to estimate adjusted relative risk ratios for the effect of education on cancer awareness and screening 16

Table S8. Two-stage least squares regression results for the relationship between education and breast cancer awareness, separately by gender in the DHS 2014 17

Table S9. Intention-to-treat regression results for the relationship between being born after the eligibility cut-off and cancer outcomes when controlling for years of schooling 18

Table S10. ITT and 2SLS regression results: the relationship of girls' education with measured cognitive skills, household wealth, and access to healthcare 19

References for Appendix 20

Supplementary Note S1. School-entry age policy

The education system in Lesotho is organized into basic education (grades 1 to 10) and secondary education (grades 11 and 12) (Raselimo and Mahao, 2015). Primary school education has been free since 2000, and attendance is high. However, fees are assessed to attend secondary school. Despite the high transition rate from primary to secondary school, many children in Lesotho do not complete secondary school, partly due to costs (UNESCO 2024). Per the Lesotho Education Act of 2010, "a parent shall enroll a learner in a primary school at the age of six years or in the year in which he or she will be six years of age by the 30th of June of that calendar year". As a result of the school-entry age policy, July-born children are on average older compared to June-born children at the beginning of grade 1. Despite starting school at older ages, July-born have completed more total years of schooling than those born in June by late adolescence and early adulthood (De Neve, Moshoeshoe and Bor 2023).

There may be several explanations for the observed differences in total educational attainment in adulthood by month of birth in Lesotho. For example, late school entrants are old-for-grade and may be more cognitively and non-cognitively mature compared to their younger classmates despite no differences in innate ability. Because of these differences in maturity and/or self-confidence in school, late entrants into primary school may accumulate more human capital (Peña PA 2017). These differences in human capital trajectories by month of birth may be further accentuated in the context of Lesotho where class sizes are large, there is considerable heterogeneity in skills in school, and there are considerable opportunity costs to schooling. For example, school-aged children frequently herd livestock, provide child and elder care, or perform other home production activities. Households and teachers may also invest more in old-for-grade children based on their perceived school performance (rather than actual performance), ultimately leading to real differences in human capital acquisition.

Supplementary Note S2. Assumptions for causal inference

We determined four assumptions that must be satisfied to have a causal conclusion (Angrist et al. 1996, Bärnighausen et al. 2017), Firstly, the instrument must have an effect on schooling. This is explored in our first stage analysis where we show a strong positive relationship between exposure to the school-entry policy cut-off and total years of schooling (**Figure 2** and **Table 3**). Secondly, the instrument must be independent of unobserved confounders conditional on observed covariates (such as year of birth and period). This implies that women born before and after June 30th were similar after controlling flexibly for year of birth and period effects. In sensitivity analyses, we included additional control variables and restricted the sample to women born 3 months before and after the cutoff (as opposed to 6 months before and after the cutoff) to further maximize the comparability of early starters and late starters on observed and unobserved characteristics (**Tables S2-S7**). The availability of two survey years also allows us to generate variation in age for a given birth cohort so that we can address age effects by simultaneously incorporating year of birth and survey year in the analysis. We also conducted a placebo test where we plotted measured adult height by women's month of birth and found that adult height is similar for women born before and after the cut-off (**Figure S3**).

Third, we assume that the eligibility cut-off affected cancer-related outcomes only through changes in years of schooling (exclusion restriction). This seems plausible given that the policy is a supply-side intervention that would not have specifically affected exposed birth months except through changes in schooling for women born after the cut-off. Additionally, we demonstrate in a regression context that after controlling for total years of schooling, the relationship between being born after the eligibility cut-off and cancer outcomes is attenuated (**Table S9**). Fourth, in order to understand our findings as the effects on those who fully adhered to the treatment (referred to as local average treatment effects), we make an assumption of monotonicity. This assumption implies that exposure to the eligibility cut-off solely resulted in individuals obtaining more education or experiencing no change in their educational attainment, without causing some individuals to obtain less education. In **Table S1**, we show the relationship between month of birth and the probability of having completed at least 1, 2, 3, … or 12 total years of schooling. Increases in educational attainment among exposed individuals appeared at all levels (years) of schooling, consistent with the monotonicity assumption.

Supplementary Note S3. Results for the subsample of men

We analyzed male respondents with data on breast cancer awareness in the DHS surveys. Data was available for men's awareness of breast cancer and men's perception towards sex affected by breast cancer in the Lesotho DHS 2014 (MOHSW/Lesotho and ICF Macro. 2010; MOHSW/Lesotho and ICF Macro. 2016). Data on breast cancer awareness was not available in the DHS 2009-10 and data on cervical cancer awareness was not available among men in both the DHS 2009-10 and DHS 2014. We defined a binary indicator for men's perception towards sex affected by breast cancer. Respondents who selected "both sexes affected" were labeled as having accurate knowledge of breast cancer; respondents who selected "only female" or "only male" were labeled as not having accurate knowledge. In OLS regression models, we observed a positive association between educational attainment and awareness towards breast cancer, as well as between education and knowledge accuracy. In 2SLS models, however, the effect of male education on breast cancer awareness did not reach conventional levels of statistical significance (i.e., $p < 0.05$) (**Table S8**).

Figure S1. Study participant flow diagram

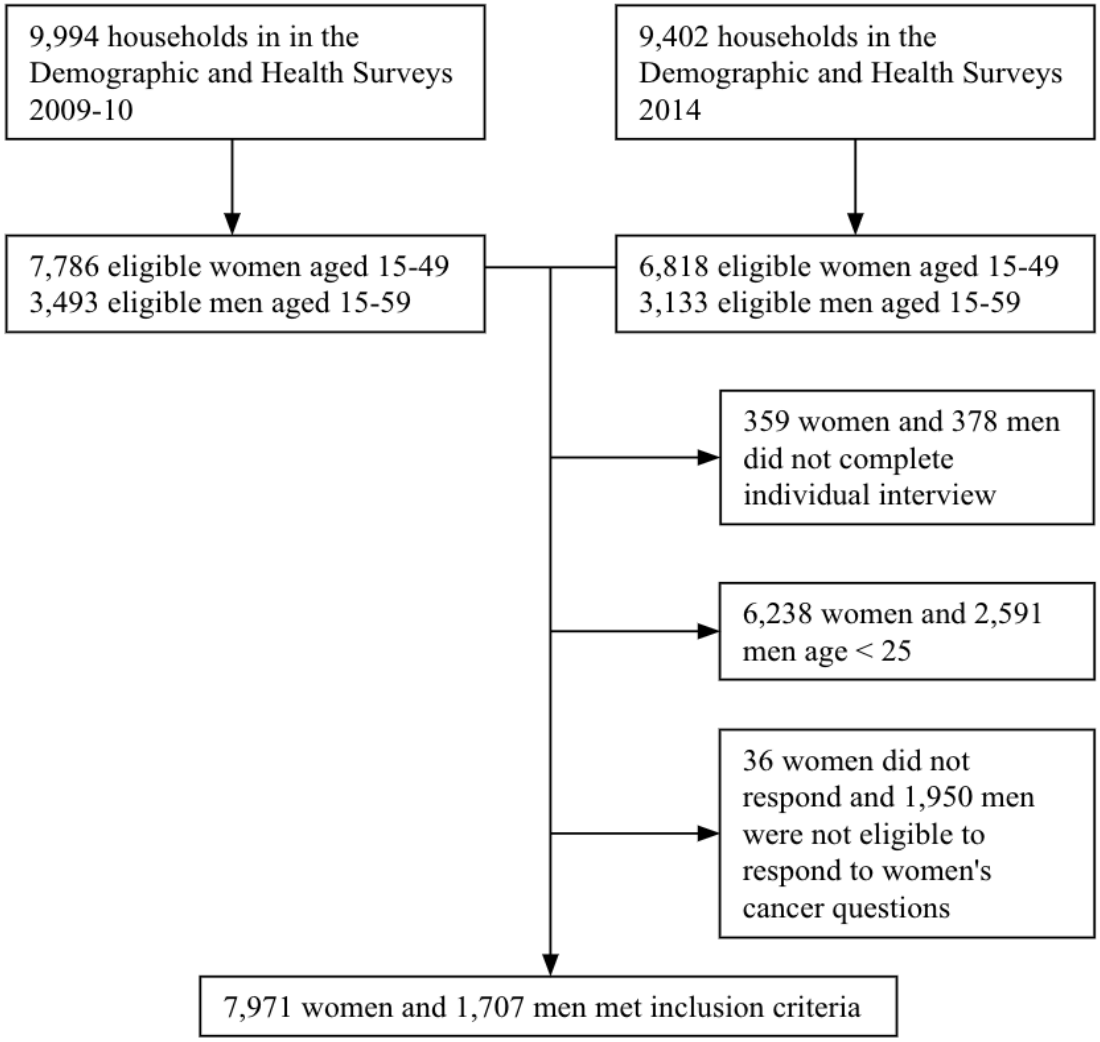

Figure S2. Distribution of month of birth among women

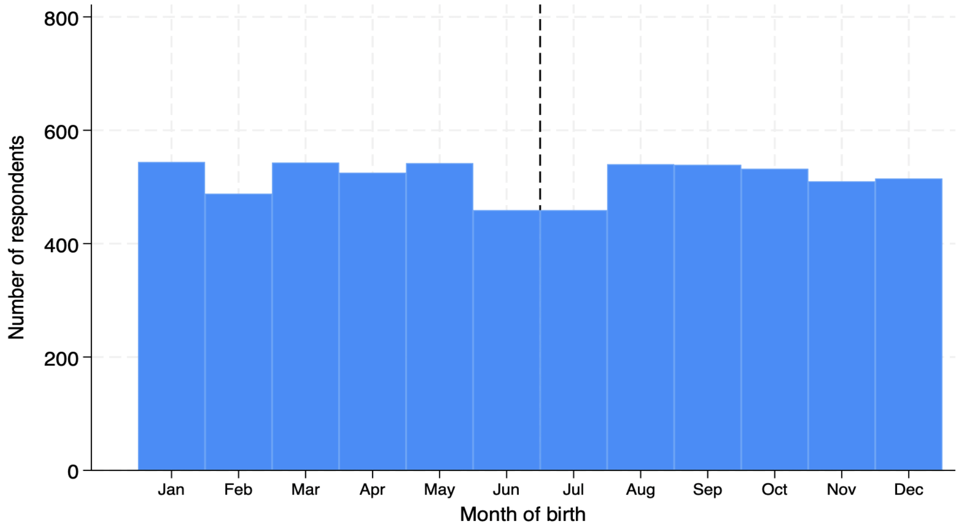

Notes: Figure shows the distribution of month of birth among women aged 15–24 years in the Demographic and Health Surveys (DHS) 2009-10 and 2014 data (*N=6,238)*. The dashed vertical line represents the school-entry age cut-off for primary school on June 30th in Lesotho. Source: Lesotho DHS 2009-10 and 2014.

Figure S3. Placebo outcome: measured adult height (in cm) by month of birth among women aged 25–49 years in the Lesotho DHS 2009-10 and 2014

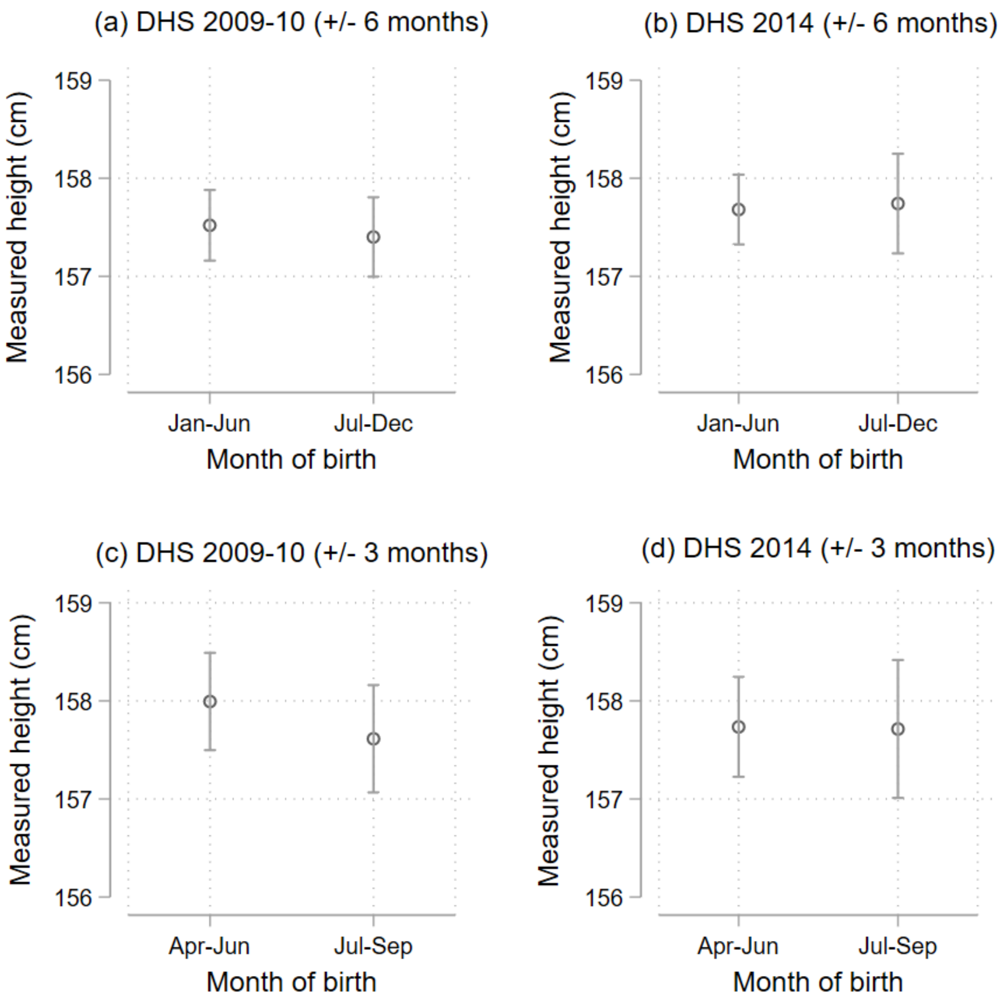

Notes: Figure shows measured adult height (in cm) by month of birth among women aged 25–49 years with 95% confidence intervals, separately by survey year. Unweighted. The sample in figures (a) and (b) includes all women born between January and December (*N=7,971*). The sample in figures (c) and (d) includes all women born between April and September (*N*=3,988). Source: data from Lesotho Demographic and Health Surveys (DHS) 2009-10 and 2014. *N=7,971.*

Figure S4. Intention-to-treat results: cancer awareness and screening by month of birth among respondents aged 25–49 years in the Lesotho DHS 2009-10 and 2014

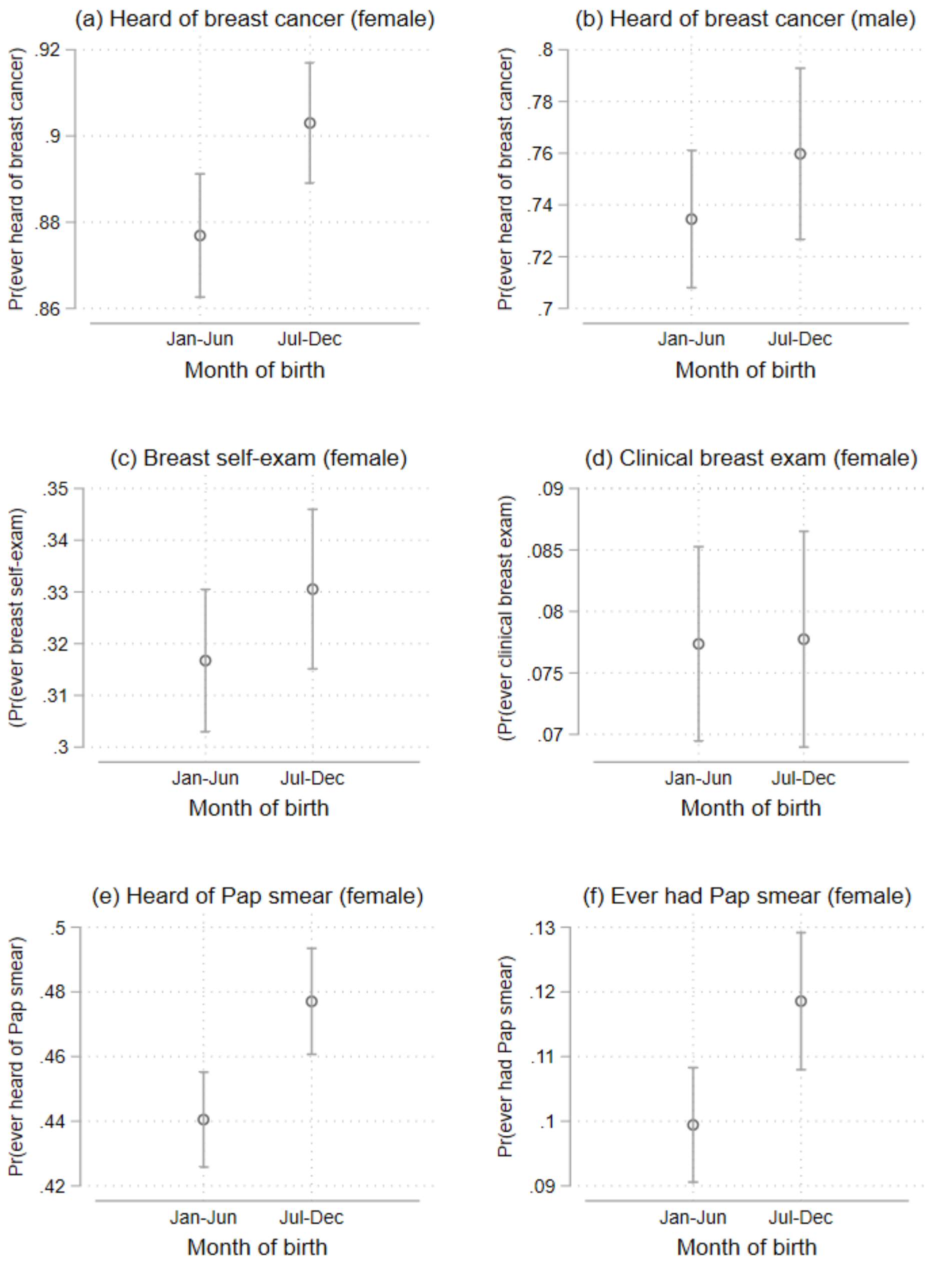

Notes: Figures (a) and (b) show the probability of having heard of breast cancer among women and men, respectively. Figures (c) and (d) show the probability of having performed either a breast self-exam or a clinical breast exam among women, respectively. Figure (e) shows the probability of ever having heard of Pap smear among women and figure (f) shows the probability of ever having received a Pap smear among women. All data are probabilities with 95% confidence intervals. Sample includes all respondents aged 25–49 years. Women born in July-December had on average higher knowledge of breast and cervical cancer (a, e) and more commonly screened for cancer (b, f) with the exception of a clinical breast exam (d). Results for men are shown in (b). Unweighted. Source: data from Lesotho DHS 2009-10 and 2014. *N=7,971.*

Figure S5. Intention-to-treat results: measured literacy, wealth and access to care by month of birth among women aged 25–49 years in the Lesotho DHS 2009-10 and 2014

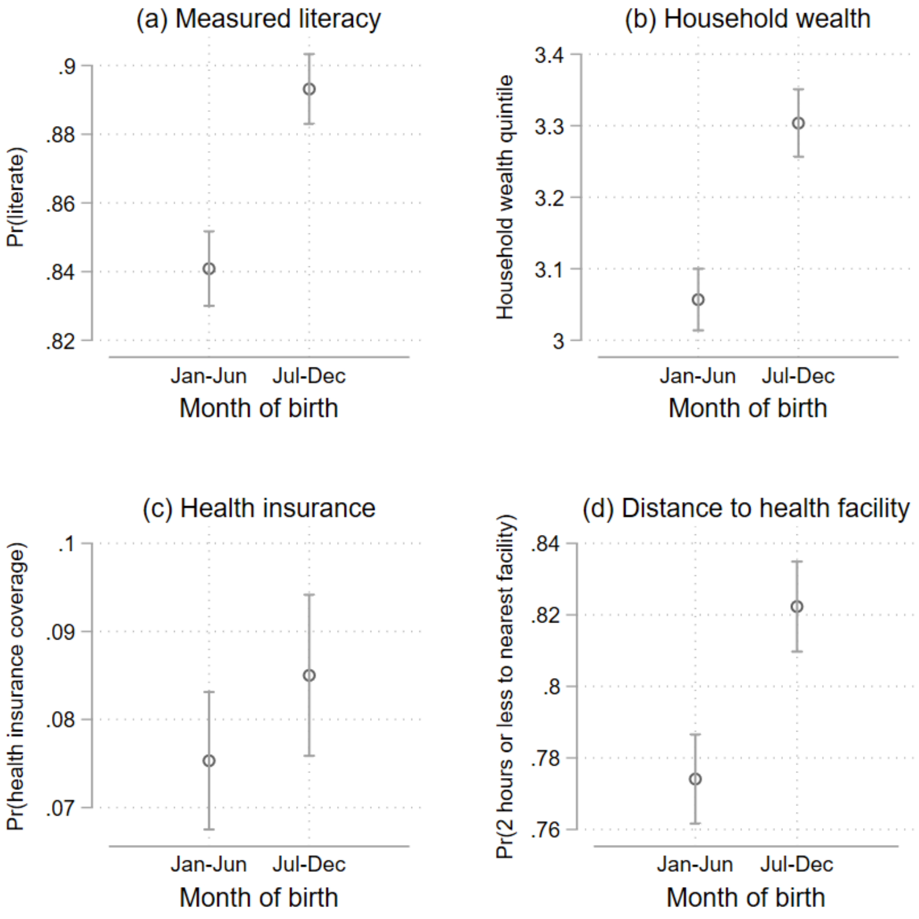

Notes: Figure shows measured literacy, household wealth, insurance coverage, and distance to care by month of birth among women aged 25–49 years with 95% confidence intervals. Women born in July-December had on average higher measured literacy (a), lived in wealthier households (b), were more likely to be enrolled in health insurance (c), and lived closer to a health facility (d). Literacy was defined by the DHS as a binary indicator which equals one if the respondent could read a whole or part of a sentence or attended secondary school or higher; and zero otherwise. Distance to health facility was defined as a binary indicator which equals one if the respondent lived within 2 hours travel time of a health facility; and zero otherwise. Unweighted. Source: data from Lesotho DHS 2009-10 and 2014. *N=7,971.*

Table S1. Intention-to-treat regression results for the relationship between being born after June 30th and year of schooling completed among women

Predictor: born between July and December (1=yes, 0=no)	Coef	*p*-value	Mean DV, January-June birth cohorts	R-squared	N
Dependent variable (DV)					
At least 1 year (1=yes)	0.013	< 0.001	0.971	0.009	7,971
	[0.007 0.020]				
At least 2 years (1=yes)	0.016	< 0.001	0.965	0.012	7,971
	[0.009 0.023]				
At least 3 years (1=yes)	0.020	< 0.001	0.952	0.012	7,971
	[0.012 0.029]				
At least 4 years (1=yes)	0.032	< 0.001	0.926	0.018	7,971
	[0.022 0.042]				
At least 5 years (1=yes)	0.046	< 0.001	0.879	0.025	7,971
	[0.033 0.059]				
At least 6 years (1=yes)	0.065	< 0.001	0.805	0.026	7,971
	[0.049 0.081]				
At least 7 years (1=yes)	0.084	< 0.001	0.668	0.033	7,971
	[0.065 0.104]				
At least 8 years (1=yes)	0.087	< 0.001	0.367	0.031	7,971
	[0.066 0.109]				
At least 9 years (1=yes)	0.077	< 0.001	0.291	0.026	7,971
	[0.056 0.098]				
At least 10 years (1=yes)	0.046	< 0.001	0.208	0.022	7,971
	[0.028 0.065]				
At least 11 years (1=yes)	0.034	< 0.001	0.161	0.021	7,971
	[0.017 0.051]				
At least 12 years (1=yes)	0.023	0.002	0.113	0.012	7,971
	[0.009 0.038]				

Notes: Table shows ordinary least regression (OLS) results for the relationship between being born after June 30th (binary) and an indicator for having completed at least X total years of schooling, estimated separately for having completed at least 1, 2, … , and 12 years of schooling. All models controlled for year of birth and survey year. We show 95% confidence intervals (CI) in brackets and two-tailed p-values for all analyses. No multiple comparisons adjustments were made. The sample includes all women ages 25-49 years in the Lesotho DHS 2009-10 and 2014 (*N*=7,971).

Table S2. Sensitivity analyses: 2SLS regression results among women for breast cancer awareness when using alternative model and sample specifications

Dependent variable (DV)	Ever heard of breast cancer (1=yes, 0=no)					
Model specification	Coef	95% CI	*p*-value	Mean DV, January-June birth cohorts	F-statistic	N
1: Using sample ages 15-49 years old	0.070	[0.003 0.136]	0.041	0.841	14.7	6.585
2: Using sample ages 35-49 years old	0.045	[-0.006 0.096]	0.081	0.887	13.1	1,792
3: Using only 2014 survey year	0.047	[0.010 0.085]	0.014	0.877	25.9	3,763
4: Using only 2009-10 survey year	n/a	n/a	n/a	n/a	n/a	n/a
5: Restricting to +/-3 months around cut-off	0.037	[-0.010 0.083]	0.124	0.875	16.3	1,923
6: Controlling for interviewer fixed effects	0.042	[0.001 0.083]	0.043	0.877	20.2	3,763

Notes: Models 1-6 are 2-stage least squares (2SLS) linear probability models in which exposure to increased schooling from the school entry age policy was used as an instrumental variable (IV) for the respondent's duration of schooling (in years). The dependent variable (DV) was a binary indicator for having ever heard of breast cancer. All models controlled for survey year and indicators for year of birth. Model 1 includes all women aged 15 – 49 years and model 2 includes all women aged 35 – 49 years (as opposed to all women aged 25 – 49 years). Model 5 includes all respondents born between April and September (as opposed to January and December). Model 6 additionally controls for an indicator for interviewer ID. We show 95% confidence intervals (CI) and two-tailed p-values for all analyses. No multiple comparisons adjustments were made. Source: Lesotho DHS 2009-10. Data on breast cancer awareness was not available (n/a) in the Lesotho DHS 2009-10.

Table S3. Sensitivity analyses: 2SLS regression results among women for breast self-exam when using alternative model and sample specifications

Dependent variable (DV)	Ever performed a breast self-exam (1=yes, 0=no)					
Model specification	Coef	95% CI	*p*-value	Mean DV, January-June birth cohorts	F-statistic	N
1: Using sample ages 15-49 years old	0.014	[-0.033 0.061]	0.553	0.304	45.3	14,167
2: Using sample ages 35-49 years old	0.023	[-0.022 0.068]	0.321	0.305	37.8	3,841
3: Using only 2014 survey year	0.027	[-0.029 0.084]	0.338	0.383	25.9	3,763
4: Using only 2009-10 survey year	0.011	[-0.034 0.056]	0.638	0.260	37.3	4,208
5: Restricting to +/-3 months around cut-off	0.017	[-0.035 0.070]	0.522	0.320	28.3	3,988
6: Controlling for interviewer fixed effects	0.018	[-0.020 0.056]	0.349	0.317	53.1	7,971

Notes: Models 1-6 are 2-stage least squares (2SLS) linear probability models in which exposure to increased schooling from the school entry age policy was used as an instrumental variable (IV) for the respondent's duration of schooling (in years). The dependent variable (DV) was a binary indicator for having ever performed a breast self-exam. All models controlled for survey year and indicators for year of birth. Model 1 includes all women aged 15 – 49 years and model 2 includes all women aged 35 – 49 years (as opposed to all women aged 25 – 49 years). Model 5 includes all respondents born between April and September (as opposed to January and December). Model 6 additionally controls for an indicator for a woman's interviewer ID. We show 95% confidence intervals (CI) and two-tailed p-values for all analyses. No multiple comparisons adjustments were made. Source: Lesotho DHS 2009-10 and 2010.

Table S4. Sensitivity analyses: 2SLS regression results among women for breast clinical screening when using alternative model and sample specifications

Dependent variable (DV)	Ever performed a breast clinical exam (1=yes, 0=no)					
Model specification	Coef	95% CI	*p*-value	Mean DV, January-June birth cohorts	F-statistic	N
1: Using sample ages 15-49 years old	-0.001	[-0.027 0.025]	0.944	0.676	45.3	14,167
2: Using sample ages 35-49 years old	0.001	[-0.025 0.026]	0.968	0.678	37.8	3,841
3: Using only 2014 survey year	-0.018	[-0.056 0.020]	0.346	0.110	25.9	3,763
4: Using only 2009-10 survey year	0.011	[-0.012 0.034]	0.355	0.049	37.3	4,208
5: Restricting to +/-3 months around cut-off	0.013	[-0.017 0.044]	0.393	0.073	28.3	3,988
6: Controlling for interviewer fixed effects	-0.006	[-0.029 0.017]	0.589	0.078	53.1	7,971

Notes: Models 1-6 are 2-stage least squares (2SLS) linear probability models in which exposure to increased schooling from the school entry age policy was used as an instrumental variable (IV) for the respondent's duration of schooling (in years). The dependent variable (DV) was a binary indicator for having ever performed a clinical breast exam. All models controlled for survey year and indicators for year of birth. Model 1 includes all women aged 15 – 49 years and model 2 includes all women aged 35 – 49 years (as opposed to all women aged 25 – 49 years). Model 5 includes all respondents born between April and September (as opposed to January and December). Model 6 additionally controls for an indicator for a woman's interviewer ID. We show 95% confidence intervals (CI) and two-tailed p-values for all analyses. No multiple comparisons adjustments were made. Source: Lesotho DHS 2009-10 and 2010.

Table S5. Sensitivity analyses: 2SLS regression results among women for cervical cancer awareness when using alternative model and sample specifications

Dependent variable (DV)	Ever heard of pap smear (1=yes, 0=no)					
Model specification	Coef	95% CI	*p*-value	Mean DV, January-June birth cohorts	F-statistic	N
1: Using sample ages 15-49 years old	0.065	[0.019 0.110]	0.005	0.350	45.3	14,167
2: Using sample ages 35-49 years old	0.078	[0.032 0.125]	0.001	0.442	37.8	3,841
3: Using only 2014 survey year	0.046	[-0.009 0.101]	0.100	0.539	25.9	3,763
4: Using only 2009-10 survey year	0.065	[0.018 0.112]	0.007	0.356	37.3	4,208
5: Restricting to +/-3 months around cut-off	0.051	[-0.002 0.104]	0.061	0.452	28.3	3,988
6: Controlling for interviewer fixed effects	0.048	[0.011 0.086]	0.011	0.441	53.1	7,971

Notes: Models 1-6 are 2-stage least squares (2SLS) linear probability models in which exposure to increased schooling from the school entry age policy was used as an instrumental variable (IV) for the respondent's duration of schooling (in years). The dependent variable (DV) was a binary indicator for having ever heard of a pap smear. All models controlled for survey year and indicators for year of birth. Model 1 includes all women aged 15 – 49 years and model 2 includes all women aged 35 – 49 years (as opposed to all women aged 25 – 49 years). Model 5 includes all respondents born between April and September (as opposed to January and December). Model 6 additionally controls for an indicator for a woman's interviewer ID. We show 95% confidence intervals (CI) and two-tailed p-values for all analyses. No multiple comparisons adjustments were made. Source: Lesotho DHS 2009-10 and 2010.

Table S6. Sensitivity analyses: 2SLS regression results among women for cervical cancer screening when using alternative model and sample specifications

Dependent variable (DV)	Ever performed pap smear (1=yes, 0=no)					
Model specification	Coef	95% CI	*p*-value	Mean DV, January-June birth cohorts	F-statistic	N
1: Using sample ages 15-49 years old	0.040	[0.014 0.067]	0.003	0.667	45.3	14,167
2: Using sample ages 35-49 years old	0.049	[0.015 0.082]	0.004	0.119	37.8	3,841
3: Using only 2014 survey year	0.056	[0.015 0.098]	0.008	0.127	25.9	3,763
4: Using only 2009-10 survey year	0.017	[-0.010 0.044]	0.226	0.076	37.3	4,208
5: Restricting to +/-3 months around cut-off	0.032	[-0.003 0.067]	0.075	0.100	28.3	3,988
6: Controlling for interviewer fixed effects	0.034	[0.009 0.060]	0.009	0.099	53.1	7,971

Notes: Models 1- 6 are 2-stage least squares (2SLS) linear probability models in which exposure to increased schooling from the school entry age policy was used as an instrumental variable (IV) for the respondent's duration of schooling (in years). The dependent variable (DV) was a binary indicator for having ever performed a pap smear. All models controlled for survey year and indicators for year of birth. Model 1 includes all women aged 15 – 49 years and model 2 includes all women aged 35 – 49 years (as opposed to all women aged 25 – 49 years). Model 5 includes all respondents born between April and September (as opposed to January and December). Model 6 additionally controls for an indicator for a woman's interviewer ID. We show 95% confidence intervals (CI) and two-tailed p-values for all analyses. No multiple comparisons adjustments were made. Source: Lesotho DHS 2009-10 and 2010.

Table S7. Sensitivity analyses: using Poisson regression models to estimate adjusted relative risk ratios for the effect of education on cancer awareness and screening

Dependent variable (DV)	Ever heard of breast cancer (1=yes, 0=no)	Breast self exam (1=yes, 0=no)	Breast clinical exam (1=yes, 0=no)	Ever heard of Pap smear (1=yes, 0=no)	Performed Pap smear (1=yes, 0=no)
	RR	RR	RR	RR	RR
Coefficient on years of schooling	1.056	1.052	0.986	1.185	1.426
95% CI	[1.012 1.102]	[0.933 1.186]	[0.734 1.323]	[1.075 1.306]	[1.069 1.904]
p-value	0.012	0.410	0.923	0.001	0.016
Mean DV, January-June birth cohorts	0.877	0.320	0.078	0.444	0.100
Observations	3,763	7,971	7,971	7,971	7,971

Notes: Adjusted relative risk ratios (RR) from multivariable Poisson regression models with 95% with confidence intervals (IVpoisson). All models control for year of birth and survey year. Exposure to increased schooling from the school-entry age policy was used as an instrumental variable for the respondent's duration of schooling (in years). We show 95% confidence intervals (CI) and two-tailed p-values for all analyses. No multiple comparisons adjustments were made. The sample included all women aged between 25-49 years in the Lesotho DHS 2009-10 and 2014. Data on breast cancer awareness was not available in the DHS 2009-10 survey.

Table S8. Two-stage least squares regression results for the relationship between education and breast cancer awareness, separately by gender in the DHS 2014

Dependent variable (DV)	Ever heard of breast cancer (1=yes, 0=no)	Ever heard of breast cancer (1=yes, 0=no)	Identifies gender affected by breast cancer (1=yes, 0=no)
	Subsample: women	Subsample: men	
Coefficient on years of schooling	0.047	0.027	0.007
95% CI	[0.010 0.085]	[-0.016 0.070]	[-0.032 0.045]
p-value	0.014	0.214	0.736
Mean DV, January-June birth cohorts	0.877	0.735	0.159
F-statistic	25.9	17.5	17.5
Observations	3,763	1,707	1,707

Notes: Table shows results from a 2-stage least squares (2SLS) linear probability model in which exposure to increased schooling from the school entry age policy was used as an instrumental variable for the respondent's duration of schooling (in years). All models controlled for survey year and indicators for year of birth. We show 95% confidence intervals (CI) and two-tailed p-values for all analyses. No multiple comparisons adjustments were made. The sample included all respondents aged between 25-59 years in the Lesotho DHS 2014. Data on accurately identifying sex affected by breast cancer was not available among women. Data on breast cancer awareness was not available among women and men in the DHS 2009-10.

Table S9. Intention-to-treat regression results for the relationship between being born after the eligibility cut-off and cancer outcomes when controlling for years of schooling

Dependent variable (DV)	Ever heard of breast cancer (1=yes, 0=no)	Breast self exam (1=yes, 0=no)	Breast clinical exam (1=yes, 0=no)	Ever heard of Pap smear (1=yes, 0=no)	Performed Pap smear (1=yes, 0=no)
	Coef	Coef	Coef	Coef	Coef
Predictor: born between July and December (1=yes, 0=no)	0.015	-0.007	-0.005	0.004	0.007
95% CI	[-0.004 0.035]	[-0.027 0.013]	[-0.017 0.007]	[-0.017 0.024]	[-0.006 0.021]
p-value	0.127	0.485	0.380	0.737	0.291
R-squared	0.046	0.060	0.020	0.154	0.075
Observations	3,763	7,971	7,971	7,971	7,971

Notes: Ordinary least squares (OLS) linear probability model after controlling for indicators for survey year, indicators for year of birth, and years of schooling completed (years). To assess whether changes in educational attainment may explain the observed relationship between exposure to the school policy and our outcomes for cancer, we ran our main ITT regression models but when additionally controlling for total years of schooling. Our hypothesis for this analysis was that if exposure to the educational policy only affected our outcomes through changes in the duration of schooling, the ITT relationship is likely attenuated when controlling for schooling. We show 95% confidence intervals (CI) and two-tailed p-values for all analyses. No multiple comparisons adjustments were made. The sample included all women aged between 25-49 years in the Lesotho DHS 2009-10 and 2014. Data on breast cancer awareness was not available in the DHS 2009-10 survey.

Table S10. ITT and 2SLS regression results: the relationship of girls' education with measured cognitive skills, household wealth, and access to healthcare

Dependent variable (DV)	Measured literacy (1=yes, 0=no)	Reads news or magazine (1=yes, 0=no)	Household wealth quintile (5=wealthiest)	Enrolled in health insurance (1=yes, 0=no)	2 hrs or less to health facility (1=yes, 0=no)
	Coef	Coef	Coef	Coef	Coef
	Model 1: ITT models controlling for period and birth cohort fixed effects				
Predictor: born between July and December (1=yes, 0=no)	0.051	0.039	0.247	0.012	0.049
95% CI	[0.036 0.065]	[0.018 0.060]	[0.183 0.311]	[-0.000 0.024]	[0.031 0.066]
p-value	< 0.001	< 0.001	< 0.001	0.055	< 0.001
R-squared	0.016	0.008	0.013	0.046	0.010
Observations	7,860	7,860	7,860	7,860	7,860
	Model 2: 2SLS models controlling for period and birth cohort fixed effects				
Coefficient on years of schooling	0.089	0.068	0.432	0.020	0.085
95% CI	[0.063 0.114]	[0.035 0.101]	[0.325 0.538]	[0.000 0.041]	[0.051 0.118]
p-value	< 0.001	< 0.001	< 0.001	0.049	< 0.001
Mean DV, January-June birth cohorts	0.841	0.306	3.06	0.076	0.774
F-statistic	62.6	62.6	62.6	62.6	62.6
Observations	7,860	7,860	7,860	7,860	7,860

Notes: Table shows intention-to-treat (ITT) and two-stage least squares regression results (2SLS) for the effect of girls' education on women's cognitive skills, household wealth, and access to healthcare. Model 1 is an ordinary least squares (OLS) linear probability model controlling for potential confounders. Model 2 is a 2-stage least squares linear probability model in which exposure to increased schooling from the school-entry age policy was used as an instrumental variable for the respondent's duration of schooling (in years). All models controlled for indicators for year of birth and survey year. We show 95% confidence intervals (CI) and two-tailed p-values for all analyses. No multiple comparisons adjustments were made. The DHS-provided wealth index is a composite measure of a household's cumulative living standard, categorizing households into quintiles (5=wealthiest). The sample included all women aged between 25-49 years in the Lesotho DHS 2009-10 and 2014.

References for Supplementary Information

Angrist JD, Imbens GW, Rubin DB (1996). Identification of causal effects using instrumental variables. *Journal of the American Statistical Association*. 91(434), 444–455.

Bärnighausen T, Oldenburg C, Tugwell P, Bommer C, Ebert C, Barreto M, Djimeu E, Haber N, Waddington H, Rockers P, Sianesi B, Bor J, Fink G, Valentine J, Tanner J, Stanley T, Sierra E, Tchetgen ET, Atun R, Vollmer S (2017. Quasi-experimental study designs series-paper 7: assessing the assumptions. *J Clin Epidemiol*. Sep;89:53-66.

De Neve JW, Moshoeshoe R, Bor J (2023). Age at School Entry and Human Capital Development: Evidence from Lesotho. *PAA Annual Meeting*. New Orleans U. S.

Ministry of Health and Social Welfare - MOHSW/Lesotho and ICF Macro (2010). Lesotho Demographic and Health Survey 2009. Maseru, Lesotho: MOHSW and ICF Macro.

Ministry of Health/Lesotho and ICF International (2016). Lesotho Demographic and Health Survey 2014. Maseru, Lesotho: Ministry of Health/Lesotho and ICF International.

Peña PA (2017). Creating winners and losers: Date of birth, relative age in school, and outcomes in childhood and adulthood. *Economics of Education Review*, 56, 152–176.

Raselimo M, Mahao M (2015). The lesotho curriculum and assessment policy: opportunities and threats. *South African Journal of Education*, 35(1), 1–12.

UNESCO (2024). UNESCO Institute for Statistics (UIS.Stat) 2024. Accessed November 1, 2024. http://data.uis.unesco.org/Index.aspx.
