# Supplementary material for: Effect of girls’ education on cancer awareness and screening in a natural experiment in Lesotho
Source: Nat Commun. 2025 Apr 20;16:3737. doi: 10.1038/s41467-025-58875-3 (PMC12009962; doi:10.1038/s41467-025-58875-3)
Supplement: Supplementary file 3 — Reporting Summary [file 41467_2025_58875_MOESM3_ESM.pdf]

Corresponding author(s): Janny Liao

Last updated by author(s): Feb 24, 2025

Reporting Summary

Nature Portfolio wishes to improve the reproducibility of the work that we publish. This form provides structure for consistency and transparency in reporting. For further information on Nature Portfolio policies, see our Editorial Policies and the Editorial Policy Checklist.

Statistics

For all statistical analyses, confirm that the following items are present in the figure legend, table legend, main text, or Methods section.

n/a	Confirmed	
☐	☒	The exact sample size (n) for each experimental group/condition, given as a discrete number and unit of measurement
☐	☒	A statement on whether measurements were taken from distinct samples or whether the same sample was measured repeatedly
☐	☒	The statistical test(s) used AND whether they are one- or two-sided<br>*Only common tests should be described solely by name; describe more complex techniques in the Methods section.*
☐	☒	A description of all covariates tested
☐	☒	A description of any assumptions or corrections, such as tests of normality and adjustment for multiple comparisons
☐	☒	A full description of the statistical parameters including central tendency (e.g. means) or other basic estimates (e.g. regression coefficient) AND variation (e.g. standard deviation) or associated estimates of uncertainty (e.g. confidence intervals)
☐	☒	For null hypothesis testing, the test statistic (e.g. F, t, r) with confidence intervals, effect sizes, degrees of freedom and P value noted<br>*Give P values as exact values whenever suitable.*
☒	☐	For Bayesian analysis, information on the choice of priors and Markov chain Monte Carlo settings
☒	☐	For hierarchical and complex designs, identification of the appropriate level for tests and full reporting of outcomes
☒	☐	Estimates of effect sizes (e.g. Cohen's d, Pearson's r), indicating how they were calculated

Our web collection on statistics for biologists contains articles on many of the points above.

Software and code

Policy information about availability of computer code

Data collection	N/A
Data analysis	Stata (version 17). All coding used in this study is available on the Harvard Dataverse repository (https://doi.org/10.7910/DVN/ATGJYB).

For manuscripts utilizing custom algorithms or software that are central to the research but not yet described in published literature, software must be made available to editors and reviewers. We strongly encourage code deposition in a community repository (e.g. GitHub). See the Nature Portfolio guidelines for submitting code & software for further information.

Data

Policy information about availability of data

All manuscripts must include a data availability statement. This statement should provide the following information, where applicable:

- Accession codes, unique identifiers, or web links for publicly available datasets
- A description of any restrictions on data availability
- For clinical datasets or third party data, please ensure that the statement adheres to our policy

We used unrestricted data which are publicly available upon request from the Demographic and Health Surveys Program (https://dhsprogram.com/). Dataset requests must include contact information, a research project title, and a description of the proposed analysis of the data.

Research involving human participants, their data, or biological material

Policy information about studies with human participants or human data. See also policy information about sex, gender (identity/presentation), and sexual orientation and race, ethnicity and racism.

Reporting on sex and gender	We determined heterogeneity by gender. To do so, we show results when stratifying our sample by gender (shown in Text S3 and Table S8 in Supplementary Information). Data was available for men's awareness of breast cancer and men's perception towards sex affected by breast cancer in the Lesotho DHS 2014 (MOHSW/Lesotho and ICF Macro. 2010; MOHSW/Lesotho and ICF Macro. 2016). Data on breast cancer awareness was not available in the DHS 2009-10 and data on cervical cancer awareness was not available among men in both the DHS 2009-10 and DHS 2014.
Reporting on race, ethnicity, or other socially relevant groupings	N/A
Population characteristics	See below in "Behavioural & social sciences study design"
Recruitment	Not applicable since we used existing secondary data from the DHS Program.
Ethics oversight	Heidelberg University Hospital Ethics Committee (S-825/2022).

Note that full information on the approval of the study protocol must also be provided in the manuscript.

Field-specific reporting

Please select the one below that is the best fit for your research. If you are not sure, read the appropriate sections before making your selection.

☐ Life sciences ☒ Behavioural & social sciences ☐ Ecological, evolutionary & environmental sciences

For a reference copy of the document with all sections, see nature.com/documents/nr-reporting-summary-flat.pdf

Behavioural & social sciences study design

All studies must disclose on these points even when the disclosure is negative.

Study description	Nationally representative quasi-experimental study using pooled cross-sectional data (quantitative).
Research sample	Data was extracted from the 2009-10 and 2014 Lesotho Demographic and Health Surveys, which are cross-sectional, individual-level surveys with data on breast and cervical cancer-related knowledge and screening practices. DHS is a two-stage cluster randomized nationally representative survey. We restricted our main analysis to female respondents ages 25 to 49.
Sampling strategy	A total of 9,994 households were invited in the 2009-10 survey and a total of 9,402 households were invited in the 2014 survey. For the 2009 survey, the individual response rates were 98% among women and 95% among men. For the 2014 survey, the individual response rates were 97% among women and 94% among men. Complete information on age, birth month, total years of schooling, and cancer-related outcomes were available for 99% of respondents, which yield a final sample of 4,208 female respondents in the 2009-10 survey, and 3,763 female respondents in the 2014 survey, and 1,707 male respondents in the 2014 survey.
Data collection	Not applicable since we used existing secondary data from the DHS.
Timing	2009-2014
Data exclusions	We excluded respondents below the age of 25 so they had the opportunity to complete their formal schooling.
Non-participation	See Figure S1 in Supplementary Information which shows a study participant flow chart.
Randomization	Not allocated into experimental groups by researchers.

Reporting for specific materials, systems and methods

We require information from authors about some types of materials, experimental systems and methods used in many studies. Here, indicate whether each material, system or method listed is relevant to your study. If you are not sure if a list item applies to your research, read the appropriate section before selecting a response.

Materials & experimental systems

n/a	Involved in the study
☒	☐ Antibodies
☒	☐ Eukaryotic cell lines
☒	☐ Palaeontology and archaeology
☒	☐ Animals and other organisms
☒	☐ Clinical data
☒	☐ Dual use research of concern
☒	☐ Plants

Methods

n/a	Involved in the study
☒	☐ ChIP-seq
☒	☐ Flow cytometry
☒	☐ MRI-based neuroimaging

Plants

Seed stocks

Report on the source of all seed stocks or other plant material used. If applicable, state the seed stock centre and catalogue number. If plant specimens were collected from the field, describe the collection location, date and sampling procedures.

Novel plant genotypes

Describe the methods by which all novel plant genotypes were produced. This includes those generated by transgenic approaches, gene editing, chemical/radiation-based mutagenesis and hybridization. For transgenic lines, describe the transformation method, the number of independent lines analyzed and the generation upon which experiments were performed. For gene-edited lines, describe the editor used, the endogenous sequence targeted for editing, the targeting guide RNA sequence (if applicable) and how the editor was applied.

Authentication

Describe any authentication procedures for each seed stock used or novel genotype generated. Describe any experiments used to assess the effect of a mutation and, where applicable, how potential secondary effects (e.g. second site T-DNA insertions, mosiacism, off-target gene editing) were examined.
